# Multivariable Regression Strength Model for Steel Fiber-Reinforced Concrete Beams under Torsion

**DOI:** 10.3390/ma14143889

**Published:** 2021-07-12

**Authors:** Ahmed F. Deifalla, Adamantis G. Zapris, Constantin E. Chalioris

**Affiliations:** 1 Department of Structural Engineering and Construction Management, Faculty of Engineering, Future University in Egypt, 90th street, New Cairo 11835, Egypt or ahmed.deifalla@fue.edu.eg; 2 Laboratory of Reinforced Concrete and Seismic Design of Structures, Civil Engineering Department, School of Engineering, Democritus University of Thrace, 67100 Xanthi, Greece; azapris@civil.duth.gr

**Keywords:** steel fiber reinforced concrete (SFRC), design, beams, torsion, analysis

## Abstract

Torsional behavior and analysis of steel fiber reinforced concrete (SFRC) beams is investigated in this paper. The purpose of this study is twofold; to examine the torsion strength models for SFRC beams available in the literature and to address properly verified design formulations for SFRC beams under torsion. A total of 210 SFRC beams tested under torsion from 16 different experimental investigations around the world are compiled. The few strength models available from the literature are adapted herein and used to calculate the torsional strength of the beams. The predicted strength is compared with the experimental values measured by the performed torsional tests and these comparisons showed a room for improvement. First, a proposed model is based on optimizing the constants of the existing formulations using multi-linear regression. Further, a second model is proposed, which is based on modifying the American Concrete Institute (ACI) design code for reinforced concrete (RC) members to include the effect of steel fibers on the torsional capacity of SFRC beams. Applications of the proposed models showed better compliance and consistency with the experimental results compared to the available design models providing safe and verified predictions. Further, the second model implements the ACI code for RC using a simple and easy-to-apply formulation.

## 1. Introduction

The addition of fibers to the concrete mass leads to the creation of fiber reinforced concrete, a composite material. Fibers are available in various shapes (straight, hooked, crimped, duoform, paddled, enlarged ends, and irregular), cross-sections (circular, rectangular, and irregular), and materials (steel, glass, or composite materials). When they are uniformly dispersed in different percentages in the concrete mix, the material properties improve. The behavior of this composite material has been extensively studied by many researchers.

### 1.1. Post-Cracking Behavior of Steel Fiber Reinforced Concrete (SFRC)

The addition of low to moderate amounts of fibers does not significantly affect the compressive strength [1,2,3,4,5,6] but leads to a less brittle failure of the compressed concrete. However, fibers have a significant effect on the strength and the post cracking behavior under tension [7,8,9]. As soon as a crack begins to form, the fibers are activated. When a crack begins to widen, it comes into contact with a number of fibers that are either perpendicular to it or positioned at an angle and resist its widening. Fibers act as crack arrestors and transmit tensile stresses through the cracks [10,11,12,13,14,15]. The gradual activation of the fibers results in the transformation of the overall behavior of the concrete from brittle to pseudo-ductile, enhance the energy dissipation capacity and reduce the width of the cracks [16,17]. The improved tensile behavior of fiber reinforced concrete leads to an enhancement of the structural element’s response under flexural, shear and torsional loading. Especially, regarding the phenomenon of torsion, previous research revealed that the behavior of an element under pure torsion is fully influenced by the behavior of the material under direct tension [18,19].

### 1.2. Behavior of SFRC Members under Torsion

Previous studies on SFRC have primarily focused on its tensile [9,20,21,22], shear [23,24,25,26,27,28,29,30,31], and flexural behavior [5,32,33,34]. While, the number of studies on SFRC’s torsional behavior remains limited, although in some load cases the torsional response of beams may govern the overall structural behavior. Since modern buildings often have complex asymmetric or even free-form design configurations, torsional actions are becoming more critical in the structural designing than ever before [35].

Torsion stiffness dramatically decreases when a reinforced concrete (RC) member experiences the first onset of torsion cracking [36,37]. This reduction in torsional stiffness is much greater than the reduction in flexural stiffness caused by flexural cracking [38]. Consequently, deformation in the form of twisting becomes more pronounced. Furthermore, inclined torsion induced cracks tend to propagate rapidly and are wider and more unsightly than flexural cracks [39,40].

The inclusion of fibers in beams subjected to torsional forces can significantly alter this mechanism, as the tensile capacity of fibers bridging the crack will improve the torsional resistance of concrete members [41,42,43]. The addition of steel fibers also provides other significant benefits, such as reduced concrete cover spalling, decreased crack width and crack spacing and, as well, deflection reduction in SFRC beams under torsion [44,45,46,47]. SFRC exhibit significantly different cracking mechanisms, enhanced structural performance and higher durability compared to plain concrete due to the superior ability of steel fibers to transfer the developed tensile stresses across cracks. Instead of forming large cracks similar to conventional RC members, SFRC structural members absorb damage through the formation of micro-cracks even at large deformations. These beneficial characteristics of SFRC inspired researchers to extensively study the effect of steel fibers on the structural performance of SFRC members with or without conventional reinforcement [27,48,49]. Relative studies revealed that steel fibers can be used as a minimum torsional reinforcement instead of conventional stirrups, making SFRC a viable alternative for torsional members that require very dense steel reinforcement [50,51,52]. This emphasizes the advantages and worthiness of SFRC applications in the structural industry when torsion occurs.

As torsion is a factor that influences structural safety, it is essential to develop an appropriate model to describe the response of members subjected to torsion in order to obtain reliable structural design when fibers are implemented in practice. Over the years several models have been developed in order to describe the torsional behavior of RC beams. Some of these models are simple and can be easily used by practice engineers, while others contain more complicated formulations but are though very accurate. Since the torsional behavior of RC members is not yet fully understood as it depends on many different factors, new models for describing the RC torsional behavior are still being proposed. In a recent study, a new plasticity based space truss model has been proposed and validated through using more than 200 experimental tests. This model uses simple equilibrium conditions but still provides a good first estimate of the ultimate torsional capacity [53]. Other recent studies have introduced models with more complicated methodologies that are able to predict the full torsional behavior of both, RC and a prestressed concrete (PC) members [54]. In order to examine the existing torsional models’ efficacy to also predict the torsional behavior of SFRC members, they should be properly modified to include the effect of the fibers. Such modifications have already been made by some researchers and few models have been proposed. These models can also be divided into two categories, the theoretical models and the simplified semiempirical models.

### 1.3. Theoretical Models Predicting the Overall Response of SFRC Members under Torsion

Some researchers have developed detailed theoretical models. These models where mainly based on the softened truss model [55] and the Bredt’s [56] thin-walled tube theory [35,57,58,59,60,61,62].

There are also some developed numerical models, as the numerical algorithm that was developed by Karayannis [63] and is a combination of the finite element and finite difference numerical techniques and can be applied in any type SFRC members of cross-sections.

Zhan and Meschke [64] also proposed a computational multilevel model that employs the implicit/explicit integration scheme and is divided in three levels of modeling, Level 1 for the pullout behavior of a single fiber, Level 2 for the crack bridging ability of fibers and Level 3 that involves the finite element method discrete crack representation model.

Hui and Lopez [65] developed a model suitable for constant section members that was based on previous formulations using average linear interpolation methods and they implemented it in finite element analysis software.

### 1.4. Simplified Models Predicting the Ultimate Torsional Strength of SFRC Members

Other researchers have focused on developing more simplified and semi-empirical models to calculate the ultimate torsional strength of SFRC elements. Mansur [66] developed a model, based on the skew bending theory, to calculate the ultimate torque moment. This model consisted of two Modes and was verified for beams under combined bending and torsion. This model was later modified to also consider the presence of shear [67]. Narayanan and Kareem-Palanjian [68] proposed two equations to predict the cracking and ultimate torsional strength of steel fiber concrete beams. The same researcher later [44] proposed a model which could calculate the ultimate torsional strength with a single expression that considers the contribution of plain concrete, the steel reinforcement and steel fibers.

Craig [69] used the theory proposed by Hsu [55] for regular RC and modified it to consider the presence of the fibers. Thus, Craig proposed two coefficients which were adopted in the expression that calculates the ultimate torsional moment. These coefficients are depended on the fiber content and type and as well the pullout strength of the fibers. Sharma [70] also extended the skew bending theory originally proposed by Hsu [55] to adapt for SFRC beams subjected to combined axial compression, bending, and torsion. Tegos [71] developed a model to predict the torsional strength of concrete beams with circular cross-sections and short discontinuous straight steel fibers that were randomly oriented and uniformly dispersed in a concrete matrix.

Nanni [72] modified the ACI 318-89 [73] code specifications for torsion design criteria for rectangular cross-section to include the fiber contribution, by adopting the approach of the fiber factor *F* proposed by Narayanan and Kareem-Palanjian [44]. El-Niema [74] also modified the ACI 318-89 [73] code and proposed an equation that considers the steel fibers contribution to the torsional strength.

Rao and Seshu [75] proposed a semi empirical formula for predicting the ultimate torsional strength of the SFRC members and Okay and Engin [76] proposed an empirical equation to predict the torque moment for SFRC beams. Amin and Bentz [77] proposed a model that modified the space truss analogy for RC members subjected to pure torsion [78] to consider the favorable contribution of fibers in the tensile response after concrete cracking. They also proposed a simplified approach of the model suitable for design.

### 1.5. Research Significance

From the literature review it is derived that in the latest studies researchers focused on developing detailed and complicated models to describe the behavior of SFRC beams under torsion. These models are accurate but are difficult to apply by practice engineers. On the other hand, there are several simplified models as described above but most of these are based on older experimental results. A common accepted model to predict the behavior of SFRC elements under torsion that is relatively simple to apply in practice at the same time accurate, does not exist. The need for establishing such a model with the potential of implementing it in modern regulations remains.

The purpose of the current study is twofold; to examine the torsion strength models for SFRC available in the literature and to address properly verified design formulations for SFRC under torsion. An experimental database with a total of 210 SFRC beams tested under torsion from 16 different experimental studies [44,46,50,57,58,68,71,74,75,76,79,80,81,82,83,84] around the world are complied. The limited existing models in the literature are adapted and further implemented to estimate the torsional strength of the tested beams. The calculated torsional strength is then compared to the experimental values obtained from the tests that were carried out. These comparisons revealed that there is still need for further improvement. Therefore, two models are proposed in the current study. The first model is based on linear regression optimization of current models from the literature. The second model is based on amending the American Concrete Institute (ACI) design code for RC members to consider the effect of steel fibers on SFRC beams’ torsional capacity.

## 2. Nonlinear Regression

### 2.1. Torsion Testing of SFRC Beams

**Figure 1:**
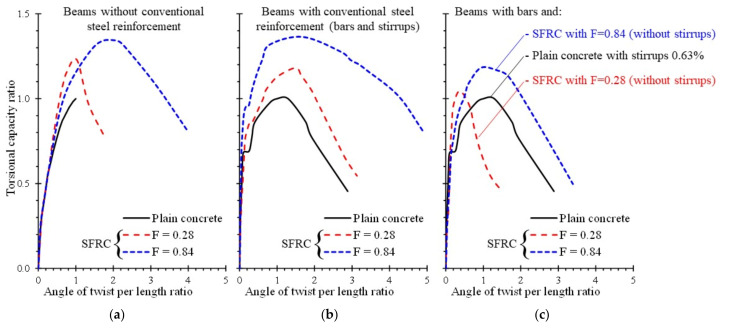
Influence of steel fibers on the torsional capacity and rotation of concrete beams: (**a**) without conventional steel reinforcement; (**b**) with full conventional steel reinforcement (bars and stirrups) and (**c**) with longitudinal bars and fibers or stirrups.

Figure 1 illustrates the experimental behavioral curves derived from torsional tests of plain concrete and SFRC beams carried out by the authors [50]. From the comparisons of Figure 1a it is clearly indicated that the use of steel fibers is essential to the beams without conventional steel reinforcement since fibers are the only reinforcement that can provide increase of the torsional strength and deformation capacity in terms of rotation, especially in the examined beam with high fiber content: *F* = 0.84, where *F* is the fiber factor *F* = *β ρ_f_ l_f_ /d_f_* that is calculated from the bond factor (*β*), the fiber volume fraction (*ρ_f_*) and the length-to-diameter or aspect ratio (*l_f_ /d_f_*). The same conclusions can be drawn from the torsional curves of beams with conventional steel reinforcement, such as longitudinal bars and transversal stirrups, shown in Figure 1b. It is also emphasized that steel fibers could be used under certain circumstances as the only shear torsional reinforcement in beams without stirrups, since experimental curves of Figure 1c indicate that the use of high fractions of steel fibers in torsional beams with only longitudinal reinforcing bars (without stirrups) provided increased strength and rotation capabilities compared to the plain concrete beams (without fibers) with both bars and stirrups.

**Figure 2:**
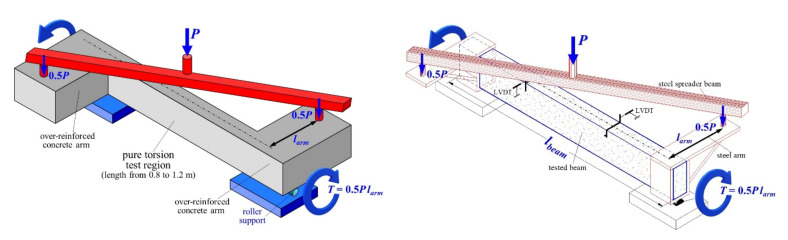
Typical experimental setup, shape and size of SFRC beams subjected torsion.

Typical experimental setup of torsional tests is shown in Figure 2 [50]. Tested beams are supported on two roller supports that ensure that the specimen is free to twist and to elongate longitudinally at both end directions during the test so as to prevent St. Venant’s effect. The load is usually applied through a diagonally placed steel spreader beam on the ends of two over-reinforced concrete or steel arms that are fixed at the end parts of each tested beam. These end parts of the specimen can bear without cracking the imposed load and, therefore, the pure torsion test region is the middle part of the beams. Rectangular, L-shape, T-shape and circular cross-sectional shapes are formed and examined in this central part of the tested beams. During the test procedure, torsional diagonal cracking and, finally, failure have been localized strictly inside this test region. The over-reinforced end parts of the beams remained quite intact. The average angle of twist per unit length of the tested beams is evaluated using the measurements of a set of linear variable displacement transducers placed at the over-hanging ends of the specimens to measure the opposite deflections of the concrete arms as the beam twisted.

Sixteen experimental studies investigated the behavior of SFRC beams under torsion since 1980’s. From the available studies, a total of 210 SFRC beams tested under torsion were collected. The experimental database was divided into two groups based on the type of reinforcement. The first category, group one, includes 123 SFRC beams without any steel reinforcement. The second group, group two, contains 87 SFRC beams with only longitudinal steel reinforcing bars or both longitudinal and transversal steel reinforcement. Table A1 and Table A2 provide an overview of the details of tested SFRC beams according to their categorization, group one, and group two.

The tested beams covered a wide range of significant parameters affecting the response. Beams with cross-sections in all shapes (rectangular, flanged, and circular), width and height, *x* and *y*, ranging from 85 mm to 300 mm, are included. Τhe cube concrete compressive strength of the beams, *f_cu_*, ranged from 14.8 MPa to 59 MPa. The fibers were hooked, duoform, crimped or plain iron wires with fiber length to diameter ratio, *l_f_/d_f_*, with a range from 37.5 to 156. The fiber volume ratio, *ρ_f_*, was between 0.3% and 6%.

In beams with both conventional reinforcement and fibers, the percentage of longitudinal bars, *ρ_l_*, ranged from 0.1% to 2% while the transversal steel reinforcement ratio, *ρ_t_*, ranged between 0% and 3%. Both the longitudinal, *f_yl_*, and transversal steel yield, *f_yt_*, range was from 250 MPa to 500 MPa.

The torsional strength, *T*, of the beams of group one ranged between 0.75 kNm and 13.58 kNm, while the torsional strength of the beams of group two ranged from 1.02 to 40.86. The minimum, maximum and average values of the abovementioned parameters of the beams that belong in group one and group two, are presented in Table 1 and Table 2, respectively.

### 2.2. Effectiveness Evaluation of Existing Models

The reliability of the models proposed by Narayanan and Kareem-Palanjian [44,68] and Tegos [71] is first examined using the experimental data collected from the literature. These models were selected because of their simplicity for design use. Although the development of these equations is mentioned in the references [44,68,71], a brief summary of the assumption is given in this study for the readers’ convenience. It is worth noting that these models were validated using experimental testing databases back in the 1980s. However, the databases that are used in the validation were limited compared to the current state of the art presented in this study. Narayanan and Kareem-Palanjian [68] proposed a formula for torsion design of SFRC rectangular beams, where the plastic torsion is calculated using the equation:

(1) $$T=0.13x^{2}y\sqrt{f_{cu}}$$

The effect of SFRC is considered using a factor (1+0.42F), so that the equation is formed as follows:

(2) $$T=0.13x^{2}y\sqrt{f_{cu}}\left(1+0.42F\right)$$

The authors extended this model to flanged beams using the well-known modification where $x^{2}y$ is replaced by $\sum x^{2}y$.

On the other hand, Tegos [71] proposed a formula for torsion design of SFRC beams with circular cross-section, where the plastic torsion is calculated using the equation:

(3) $$T=\frac{\pi }{16}D^{3}f_{t}$$

where, the $f_{t}$ is the concrete tensile strength taken as $f_{t}=0.45\sqrt{f_{cu}}$ and the beneficial effect of the added steel fibers is considered using an empirical factor. So, the equation finally takes the following form:

(4) $$T=0.09D^{3}\sqrt{f_{cu}}\left(1+\frac{{\left(\frac{l_{f}}{d_{f}}\right)}^{1.5}\rho_{f}}{15}\right)$$

According to the aforementioned models, the torsional strength (*T*) for SFRC beams without conventional steel reinforcement (group one beams) can be calculated as follows:

(5) $$T=\{\begin{array}{c} 0.13x^{2}y\sqrt{f_{cu}}\left(1+0.42F\right)\;\;\;\;\;\;\mathrm{for}\;\mathrm{rectangular}\;\mathrm{section}\\ 0.13\sum x^{2}y\sqrt{f_{cu}}\left(1+0.42F\right)\;\;\;\;\;\;\;\;\;\mathrm{for}\;\mathrm{flanged}\;\mathrm{section}\\ 0.09D^{3}\sqrt{f_{cu}}\left(1+\frac{{\left(\frac{l_{f}}{d_{f}}\right)}^{1.5}\rho_{f}}{15}\right)\;\;\;\;\mathrm{for}\;\mathrm{circular}\;\mathrm{section} \end{array}$$

where, *x* and *y* is the smaller and larger dimension of the cross-section, respectively; *f_cu_* is the cubic compressive strength of the concrete; *D* is the diameter of the cross-section; *l_f_* and *d_f_* is the length and diameter of fiber, respectively; *ρ_f_* is the volume ratio of fibers; *F* is the fiber factor, which is taken as *β (l_f_ /d_f_)* *ρ_f_* and *β* is the bond coefficient of steel fiber.

In addition, Narayanan and Kareem-Palanjian [44] proposed that the torsion strength is the superposition of three components, the torsion strength of plain concrete and two space trusses to consider the contribution of SFRC and the conventional steel reinforcements. These components are calculated using the following formulations respectively:

(6) $$0.13\sum x^{2}y\sqrt{f_{cu}}$$

(7) $$0.22F\frac{x_{0}y_{0}}{x_{0}+y_{0}}xy\sqrt{f_{cu}}$$

(8) $$k_{2}\frac{x_{1}y_{1}}{s}A_{s}f_{ty}$$

Thus, the torsional strength, *T*, of SFRC beams with only longitudinal steel reinforcing bars or both longitudinal and transversal steel reinforcement (group two beams) can be calculated by the equation:

(9) $$T=0.13\sum x^{2}y\sqrt{f_{cu}}+0.22F\frac{x_{0}y_{0}}{x_{0}+y_{0}}xy\sqrt{f_{cu}}+k_{2}\frac{x_{1}y_{1}}{s}A_{s}f_{ty}$$

where, *x*, *y*, *f_cu_* and *F* as noted above; *x*_0_ and *y*_0_ is the smaller and larger center to center dimension of the thin wall tube analogy, which is taken approximately as (5/6) *x* and (5/6) *y*, respectively; *x*_1_ and *y*_1_ is the smaller and larger dimension of the steel stirrup, which is taken approximately as 0.9 *x* and 0.9 *y*, respectively and *f_ty_* is yield stress of transversal steel reinforcement (stirrups) and *s* is the spacing between steel stirrups along the longitudinal direction of the beam. Further, *k*_2_ is the longitudinal reinforcement factor, which is calculated from the expression:

(10) $$k_{2}=\left[0.2m+\sqrt{m}\left(\frac{0.45y_{1}}{x_{1}}-\frac{s}{x_{1}+y_{1}}\right)\right]$$

where, *x*_1_ and *y*_1_ as noted above; *m* is the ratio between the longitudinal and transversal reinforcement, which is taken as *ρ_l_ f_ly_* /*ρ_t_ f_ty_*; *ρ_l_* and *ρ_t_* is longitudinal and transversal steel reinforcement ratio; *f_ly_* is yield stress of longitudinal steel reinforcing bars.

The assessment of the performance of various models is based on statistical measures including, but not limited to, the average, the coefficient of variation, and the lower 95%, which indicate the accuracy, the consistency, and the safety, respectively. These measures are applied on the torsion safety factor (TSF) as follows: (1) the closer the average is to unity, the more accurate is the model; (2) the lower the value of the coefficient of variation, the more consistent is the model; and (3) the higher value of the lower 95% limit value and above the safety factor of design codes (approximately 0.85), the safer the model is. It is the minimum TSF value obtained using the model with 95% confidence level. The confidence interval is calculated assuming a standard normal distribution. In addition, a significant level value of 0.05 represents the 95% confidence level, thus the lower 95% confidence limit is calculated using the following expression:

(11) $$\mathrm{Lower}\;95\%=\mathrm{Average}-1.96\left(\frac{\mathrm{Standard}\;\mathrm{deviation}}{\sqrt{\mathrm{number}\;\mathrm{of}\;\mathrm{samples}}}\right)$$

**Figure 3:**
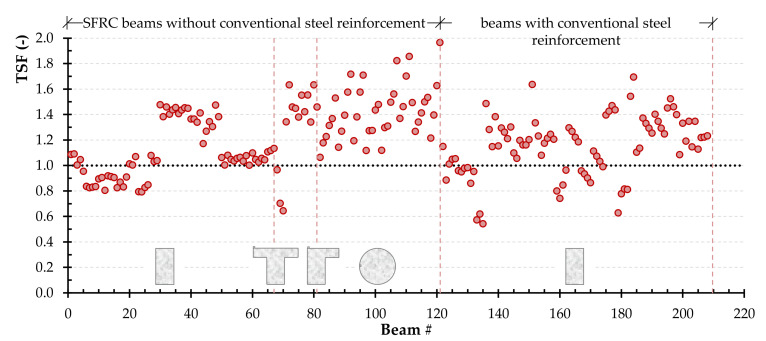
The TSF for each beam of group one (without conventional steel reinforcement) and group two (with conventional steel reinforcement) based on the beams’ cross-sectional shape (rectangular, flanged or circular) using the combined Narayanan and Kareem-Palanjian and Tegos (NKPT) model.

Such technique was applied in many previous investigations [85,86,87,88]. It is worth noting that applying the principles of reliability is out of scope of this study, it is worthy of future study [89]. It is noted that TSF is defined as the ratio between the experimentally determined torsional strength and the one calculated using the combined Narayanan and Kareem-Palanjian and Tegos (NKPT) model. The TSF value is used to evaluate the accuracy of the models. Figure 3 displays the TSF for each beam of group one and group two. The TSF for the beams of group one is further categorized based on the beams’ cross-sectional shape (rectangular, flanged or circular). The average TSF value for the beams of group one with rectangular, flanged, and circular cross-section shapes is 1.11, 1.47, and 1.42, respectively, while the coefficient of variation is 22%, 7%, and 15%. In the case of SFRC concrete beams with steel reinforcement, the average TSF is 1.14 and the coefficient of variation is 20%. It is clear that for both conventionally and non-conventionally reinforced concrete beams, the aforementioned model is relatively accurate with a reasonable average and a rather slightly higher coefficient of variation. In addition, the limit at 95% confidence level, which is commonly used to define design code specifications, was found to be 1.04, 1.41, and 1.36 for rectangular, flanged, and circular cross-section shapes, respectively, and 1.09 for the group two beams.

**Figure 4:**
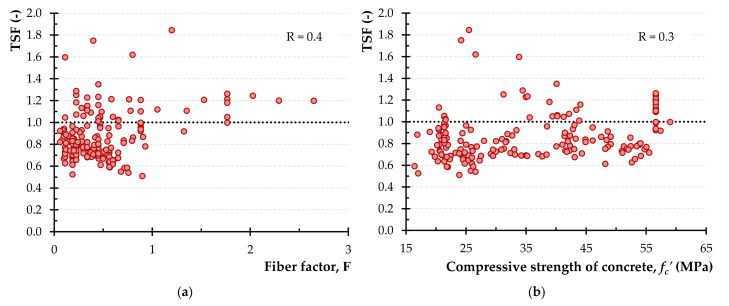

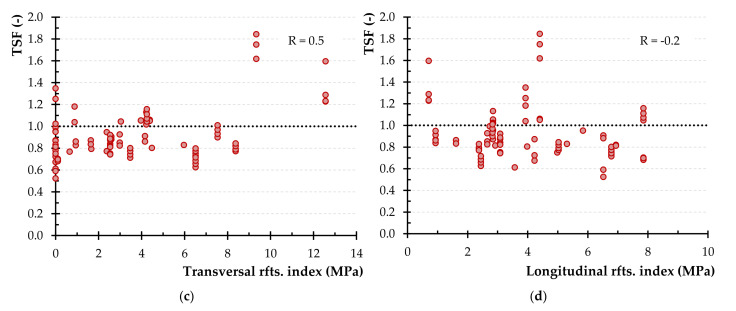
The TSF using the combined Narayanan and Kareem-Palanjian and Tegos (NKPT) model versus: (**a**) the fiber factor; (**b**) the concrete compressive strength; (**c**) the transversal reinforcement index and (**d**) the longitudinal reinforcement index.

Figure 4 illustrates the variation of the experimental-to-calculated strength ratio using the aforementioned model versus various parameters as well as the values of the correlation coefficient, R. From the diagrams of Figure 4a–d it is deduced that R values are 0.4, 0.3, 0.5 and −0.2, for the parameters *F*, *f_c_′*, the transversal and the longitudinal reinforcement index, respectively (“reinforcement” is denoted as “rfts.” in Figure 4c,d for simplicity). The rather high values of R (more than 0.3) indicate the inability of the aforementioned model to capture the effect of the examined parameters on the torsional strength of SFRC beams since TSF seems to depend on the variables of the fiber factor and the transversal reinforcement index (correlation with R > 0.3).

### 2.3. The Modified NKPT Model

In order to improve the predictions of the available models, the constants were optimized using multi-linear regression. After the optimization of the constants, the torsional strength (*T*) for SFRC beams without any steel reinforcement can be calculated as:

(12) $$T=\{\begin{array}{c} 0.19x^{2}y\sqrt{f_{cu}}\left(1+0.04F\right)\;\;\;\;\mathrm{for}\;\mathrm{rectangular}\;\mathrm{section}\\ 0.1D^{3}\sqrt{f_{cu}}\left(1+0.08F\right)\;\;\;\;\;\;\;\;\;\;\;\;\;\;\;\;\mathrm{for}\;\mathrm{circular}\;\mathrm{section}\\ 0.2\sum x^{2}y\sqrt{f_{cu}}\left(1+0.15F\right)\;\;\;\;\;\mathrm{for}\;\mathrm{flanged}\;\mathrm{section} \end{array}$$

While for beams with SFRC and conventional reinforcement, the torsional strength (*T*) can be calculated by the following expression:

(13) $$T=0.2\sum x^{2}y\sqrt{f_{cu}}+0.13F\frac{x_{0}y_{0}}{x_{0}+y_{0}}xy\sqrt{f_{cu}}+k\frac{x_{1}y_{1}}{s}A_{s}f_{ty}$$

**Figure 5:**
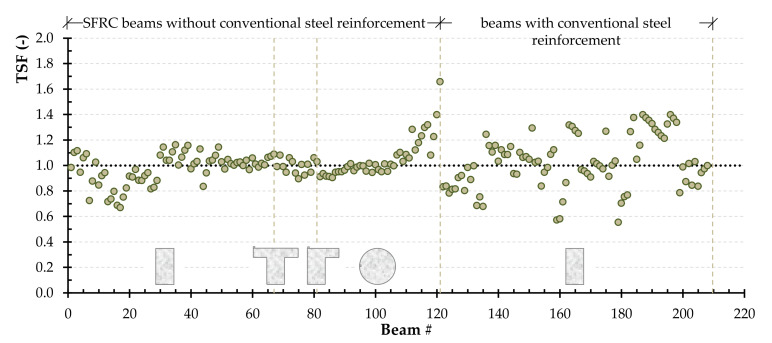
The TSF for each beam of group one (without conventional steel reinforcement) and group two (with conventional steel reinforcement) based on the beams’ cross-sectional shape (rectangular, flanged or circular) using the modified NKPT model.

Figure 5 presents the TSF values using the modified NKPT model depending on the cross-sectional shape (rectangular, flanged, and circular, respectively) for the beams of group one, and the TSF values for the beams of group two. After the constants’ optimization, the average TSF for group one beams with rectangular cross-sections is 0.96, 0.99 for those with flanged cross-sections, and 1.06 for those with circular cross-sections. While, the coefficients of variation are 14%, 6%, and 15%, respectively. The proposed model seems to be accurate in predicting torsional strength, even for beams with flanged and circular cross-sections, where the original (existing) model’s accuracy was rather limited in non-rectangular beams.

Further, the limit at 95% confidence level is calculated and the values are 0.92, 0.95, and 1.01 for the beams with rectangular, flanged, and circular cross-section shapes, respectively. The same is observed in beams of group two, with average value and coefficient of variation of TSF equal to 1.02 and 20%, respectively, while the limit at 95% confidence level value is 0.97.

**Figure 6:**
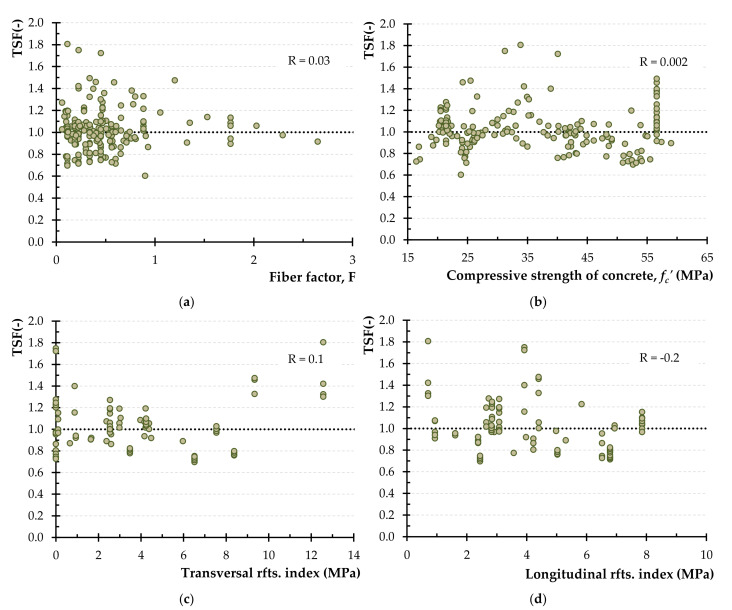
The TSF using the modified NKPT model versus: (**a**) the fiber factor; (**b**) the concrete compressive strength; (**c**) the transversal reinforcement index and (**d**) the longitudinal reinforcement index.

The torsional strength of the examined torsional beams calculated using the modified NKPT model are depicted in the diagrams of Figure 6 in terms of TSF values (ratio of the experimentally obtained strength and the calculated one) versus the parameters affecting the capacity of the SFRC beams. Based on these diagrams, the values of the correlation coefficient are R = 0.03, 0.002, 0.1 and −0.2, for the parameters *F*, *f_c_′*, the transversal and the longitudinal reinforcement index, respectively (“reinforcement” is denoted as “rfts.” in Figure 6c,d for simplicity).

The lower values of R derived from the diagrams of Figure 6a–d with respect to the corresponding R values of Figure 4a–d indicate the weak degree of association between the predictions of the proposed model in terms of TSF and the examined variables. The low values of R (less than 0.3) shown in Figure 6a–d express the ability of the proposed model to capture the effect of the examined parameters. Especially, R values are very close to 0 for the parameters of fiber factor and the concrete compressive strength, which means that TSF do not depend on these two variables. The absence of correlation between TSF and the two examined parameters suggests the safety of the proposed model to provide correct predictions.

### 2.4. The Improved ACI Formulation

Furthermore, the most recent American Concrete Institute design for torsion was adapted and properly modified to include the effect of the SFRC. In addition, multi-linear regression was used to optimize the constants in the model. Thus, the torsional strength (*T*) in case of beams with no conventional reinforcement can be calculated with the following expression:

(14) $$T=c_{1}\sqrt{f_{c}^{\prime }}\;\left(\frac{A_{cp}^{2}}{P_{cp}}\right)\left(1+c_{2}F\right)$$

where,$\;f_{c}^{\prime }$ is the cylinder compressive strength of the concrete; $A_{cp}$ is the total concrete cross-sectional area; $P_{cp}$ is the perimeter of concrete section; *F* is the fiber factor, which is taken as *β (l_f_ /d_f_)* *ρ_f_*; *β* is the bond coefficient of steel fiber and $c_{1}$ and $c_{2}$ are constants. $c_{1}$ value is equal to 0.5, 0.55 and 0.86 for beams with rectangular, flanged and circular section and $c_{2}$ value is taken as 0.04, 0.08 and 0.92, respectively.

For SFRC beams with conventional reinforcement the torsional strength (*T*) can be calculated as:

(15) $$T=\min\;\{\begin{array}{c} 1.5\frac{A_{0}A_{t}}{s}f_{ty}cot\theta \\ 1.5\frac{A_{0}A_{l}}{P_{h}}f_{ly}tan\theta \end{array}+0.6F\sqrt{f_{c}^{\prime }}\;\left(\frac{A_{cp}^{2}}{P_{cp}}\right)$$

where, *F*, $f_{c}^{\prime }$, $A_{cp}$, $P_{cp}$ as noted above; *A*_0_ is the area enclosed inside centerline of shear flow path; *A_t_* is the cross-section area of one branch of the steel stirrups; *A_l_* is the total cross-sectional area of longitudinal steel reinforcement; *s* is the spacing between steel stirrups along the beam direction; *P_h_* is the perimeter of outermost closed stirrup; *f*_ty_ and *f*_ly_ is the yield stress of transversal and longitudinal steel reinforcement, respectively and *θ* is the angle of inclination of the concrete strut, taken as 45°.

**Figure 7:**
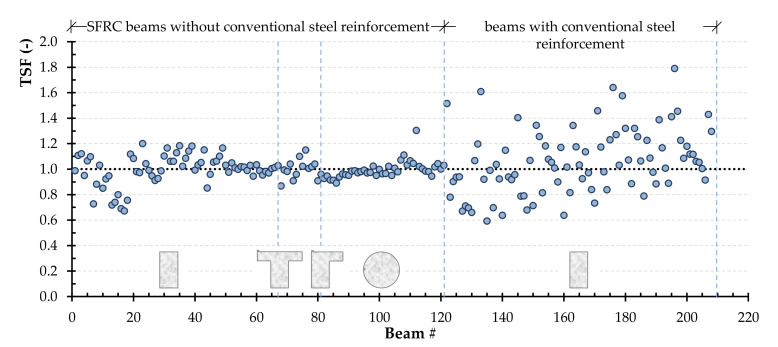
The TSF for each beam of group one (without conventional steel reinforcement) and group two (with conventional steel reinforcement) based on the beams’ cross-sectional shape (rectangular, flanged or circular) using the improved ACI formulation.

The improved ACI formulation was used to calculate the torsional strength of the tested beams. Figure 7 shows the TSF using the improved ACI formulation and that measured for tested beams of group one, whose shape is rectangular, flanged, and circular, and that of group two, respectively. The average value of TSF for the tested beams of group one was equal to 1.00, 1.01, 0.99, and that of group two 1.05, respectively. While, the coefficient of variation was 14%, 7%, 7%, and 24%, respectively. Also, the values of the limit at 95% confidence level, are 0.96, 0.97, and 0.97 for the rectangular, flanged, and circular cross-section shapes, of the group one respectively and 0.99 for the group two. It is clear that the available mode is relatively accurate with a reasonable average, however consistent with relatively lower coefficient of variation.

**Figure 8:**
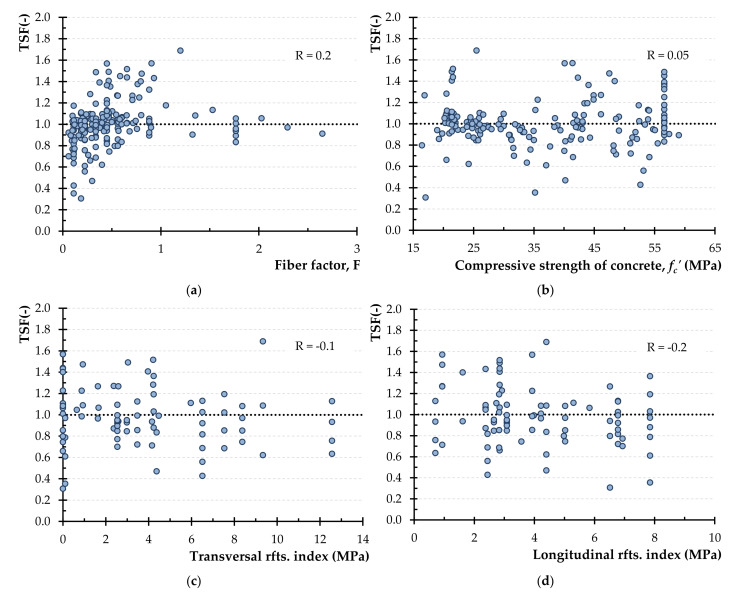
The TSF using the improved ACI formulation versus: (**a**) the fiber factor; (**b**) the concrete compressive strength; (**c**) the transversal reinforcement index and (**d**) the longitudinal reinforcement index.

Diagrams of Figure 8a–d display the variation of the TSF values derived by improved ACI formulation versus the parameters *F*, *f_c_′*, the transversal and the longitudinal reinforcement index, respectively (“reinforcement” is denoted as “rfts.” in Figure 8c,d for simplicity), along with the correlation coefficient values that equal to R = 0.2, 0.05, −0.1 and −0.2, respectively. The less than 0.3 values of R indicate the ability of the proposed model to be less dependent on these parameters and, consequently, this weak correlation expresses that it can yield safe calculations concerning the torsional strength of SFRC beams.

## 3. Comparing Various Models

Table 3 provides the overall average, the coefficient of variation, the maximum and minimum values of the ratio between the calculated torsion strength using each model and that measured one by experimental testing, in order to better compare the efficiency of the models.

The average values of the ratios using the original model, the modified NKPT model, and the improved ACI formulation are 1.21, 1.01, and 1.02, respectively, with corresponding coefficients of variance of 22%, 17%, and 19%, while the lower 95 percent TSF values were 1.17, 0.98, and 0.99, respectively.

**Figure 9:**
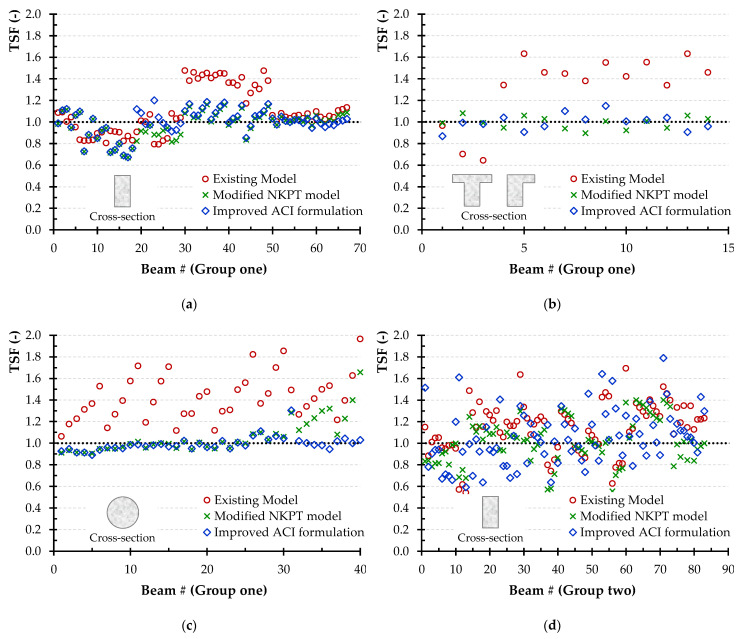
The TSF of each beam using all the models according to the group the beam belongs and the cross-section shape of the beam: (**a**) rectangular cross-section of group one; (**b**) flanged cross-section of group one; (**c**) circular cross-section of group one and (**d**) group two.

In addition, Figure 9 displays the TSF ratio values for all the tested beams using all three models’ predictions and based on the beams’ categorization and the shape of their cross-sections.

Specifically, diagrams of Figure 9a–c illustrate a comparison between the values of the TSF obtained using all three models for the beams of group one, with rectangular, flanged and circular cross-section, respectively, while Figure 9d diagram presents the TSF values of group two beams. All these diagrams indicate that both proposed models clearly present marginally better predictions than the existing one. Further, the improved ACI formulation provides more accurate predictions by simpler design calculations.

**Figure 10:**
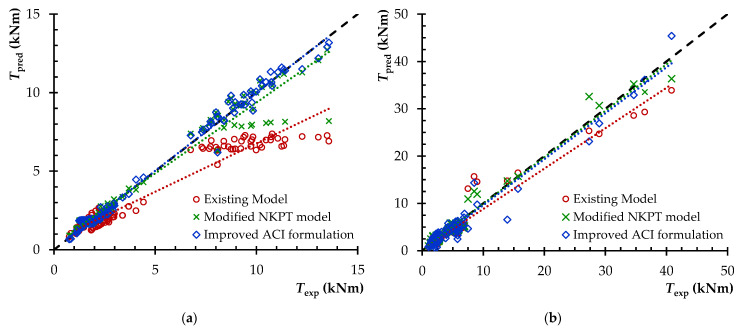
Predicted versus experimental ultimate torsion strength of the beams of: (**a**) group one and (**b**) group two.

Furthermore, for a better evaluation of the effectiveness of the examined models, Figure 10 presents the maximum torsional strength obtained from the experiments, *T_exp_*, versus the torsional strength predictions, *T_pred_*, as calculated by the three models for all SFRC beams of the database. The linear trend line for each model (dotted lines) as well as the ideal line (dashed line for *T_exp_* = *T_pred_*) are also depicted in the diagrams. In particular, in Figure 10a (beams of group one) both proposed models show a considerably higher accuracy when compared to the existing one. Further, all three models appear to be effective in estimating the maximum torsional strength for the beams of group two, as shown in Figure 10b, however, the two proposed models seem to provide more accurate predictions.

## 4. Conclusions

The following concluding remarks can be drawn from this study:

- A database of 210 SFRC beams tested under torsion from 16 different experimental studies conducted around the world is properly compiled and examined for the purposes of this research. It comprises beams with rectangular, flanged and circular cross-sections with various dimensions and shapes. Further, beams without conventional steel reinforcement, with longitudinal bars only and full torsional reinforcement (steel bars and stirrups) are included in order to establish the validity of the proposed approach based on a broad range of parametric studies. The majority of the experimental campaigns follow similar typical torsional testing setup and morphology and, therefore, test results could be considered, up to a point, comparable.
- Two models for predicting the torsional strength of SFRC beams that had been reported in the literature were adapted and their efficacy was evaluated. The predicted torsional strength is compared to the experimental values obtained from the tests. The values of the average and the coefficient of variation of the ratios between the experimentally obtained torsional strength and the calculated one revealed that further improvement and refinement of the models is still required.
- Multi-linear regression was used to optimize the constants that influence the torsional strength in order to improve the predictions of the available models. Using the optimization of the constants, the proposed model (the modified NKPT model) demonstrated particularly high accuracy in estimating torsional strength and lower coefficient of variation as compared to the original one.
- The ratio of the experimentally obtained strength and the calculated one using the examined model were correlated with four parameters affecting the torsional capacity of the SFRC beams. The values of the correlation coefficient indicated that the proposed models are less dependent on these parameters and, therefore, are capable to capture their effect on the torsional strength providing correct predictions.
- A second model is also proposed, which is based on modifying the ACI design code for RC members to account for the effect of steel fibers on the torsional capacity of SFRC beams. When compared to other design models which provide safe and validated predictions, the proposed model demonstrated higher compliance and consistency with the experimental results. Further, this model employs a simple and easy-to-apply formulation to implement the ACI code for RC. The developed optimizing methodology could be enriched later with further experimental tests that will assist in the refinement of the proposed models.

## Figures and Tables

**Table 1:** The minimum, maximum and average values of the significant parameters of the tested SFRC beams of group one.

Value	*x*	*y*	*β*	*ρ* *_f_*	*l* *_f_* */d* *_f_*	*f_cu_*	*T*
	(mm)	(mm)		(%)		(MPa)	(kNm)
Minimum	85	85	0.5	0.3	37.5	14.8	0.75
Maximum	250	250	1.3	6.0	156	59.0	13.58
Average	153	209	0.6	1.5	62.9	33.9	5.03

**Table 2:** The minimum, maximum and average values of the significant parameters of the tested SFRC beams of group two.

Value	*x*	*y*	*β*	*ρ_f_*	*l_f_/d_f_*	*f_cu_*	*f_yl_*	*ρ_l_*	*f_yt_*	*ρ_t_*	*T*
	(mm)	(mm)		(%)		(MPa)	(MPa)	(%)	(MPa)	(%)	(kNm)
Minimum	85	85	0.5	0.3	37.5	16.4	250	0.14	250	0.00	1.02
Maximum	300	310	1.0	3.0	100	55.5	500	1.61	500	2.51	40.86
Average	116	201	0.5	0.9	74.3	37.4	411	0.99	420	1.03	5.77

**Table 3:** The TSF values for existing and proposed models.

Statistical Measures	Existing Model	Modified NKPT Model	Improved ACI Formulation
Maximum	1.97	1.66	1.79
Minimum	0.54	0.55	0.59
Average	1.21	1.01	1.02
Coefficient of variation	22%	17%	19%
Lower 95%	1.17	0.98	0.99

## Data Availability

Data available on request.

## Glossary

*β*	The bond coefficient of steel fiber.
*θ*	The angle of inclination of the concrete strut, taken as 45°.
*ρ_f_*	The volume ratio of fibers.
*ρ_l_*	Longitudinal steel reinforcement ratio.
*ρ_t_*	Transversal steel reinforcement ratio.
*A_cp_*	Total concrete cross-sectional area.
*A_l_*	Total cross-sectional area of longitudinal steel reinforcement.
*A*_0_	The area enclosed inside centerline of shear flow path.
*A_s_*	Cross-sectional area of steel stirrup.
*A_t_*	The cross-section area of one branch of the steel stirrups.
*c*_1_	Constant with value equal to 0.5, 0.55 and 0.86 for beams with rectangular, flanged and circular cross-section, respectively.
*c*_2_	Constant with value equal to 0.04, 0.08 and 0.92 for beams with rectangular, flanged and circular cross-section, respectively.
*D*	The diameter of cross-section.
*d_f_*	Diameter of fiber.
*F*	The fiber factor, which is taken as *β (l_f/_d_f_) ρ_f_*.
$f_{c}^{\prime }$	The cylinder compressive strength of the concrete.
$f_{cu}$	The cubic compressive strength of the concrete.
$f_{ly}$	The yield stress of longitudinal steel reinforcing bars.
$f_{t}$	The concrete tensile strength, taken as $\left(0.45\sqrt{f_{cu}}\right)$.
$f_{ty}$	The yield stress of transversal steel reinforcement (stirrups).
*k*_2_	The longitudinal reinforcement factor.
*l_f_*	Length of fiber.
*m*	The ratio between the longitudinal and transversal reinforcement, which is taken as *ρ_l_ f_ly/_ρ_t_ f_ty_*.
*P_cp_*	The perimeter of concrete section.
*P_h_*	The perimeter of outermost closed stirrup.
*s*	The spacing between steel stirrups along the longitudinal direction of the beam.
*T*	Torsion capacity of the cross-section.
*x*	the smaller dimension of the cross-section.
*x*_0_	The smaller center to center dimension of the thin wall tube analogy, which is taken approximately as (5/6) *x*.
*x*_1_	The smaller dimension of the steel stirrup, which is taken approximately as 0.9 *x.*
*y*	The larger dimension of the cross-section.
*y*_0_	The larger center to center dimension of the thin wall tube analogy, which is taken approximately as (5/6) *y*.
*y*_1_	The larger dimension of the steel stirrup, which is taken approximately as 0.9 *y*.

## Appendix A

**Table A1:** Details of the tested SFRC beams of group one (123 specimens).

Reference	Name	Shape ^1^	*x*	*y*	Type of Fibers ^2^	*β*	*ρ_f_*	*l_f_/d_f_*	*f_cu_*	*T*
			(mm)	(mm)			(%)		(MPa)	(kNm)
[68]	A-1-1	R	85	178	DF	0.91	1.00	97	56.6	1.87
	A-1-1-5	R	85	178	DF	0.91	1.50	97	57.4	2.15
	A-1-2	R	85	178	DF	0.91	2.00	97	59.0	2.24
	B-1-1	R	85	178	DF	0.91	1.00	97	38.5	1.49
	B-1-2	R	85	178	DF	0.91	2.00	97	39.4	1.74
	B-1-3	R	85	178	DF	0.91	3.00	97	56.6	2.22
	B-2-2	R	85	178	DF	0.78	2.00	49	56.6	1.37
	B-2-4	R	85	178	DF	0.78	4.00	49	56.6	1.71
	B-2-6	R	85	178	DF	0.78	6.00	49	56.6	2.06
	B-3-1	R	85	178	DF	1.30	1.00	81	56.6	1.62
	B-4-1	R	85	178	DF	1.30	1.00	104	56.6	1.78
	B-5-1	R	85	178	DF	1.30	1.00	156	56.6	1.87
	B-6-1	R	85	178	RF	0.50	1.00	75	56.6	1.34
	B-7-1	R	85	178	RF	0.50	1.00	97	56.6	1.38
	B-8-1	R	85	178	RF	0.75	1.00	104	56.6	1.51
	B-9-1	R	85	178	CF	0.75	1.00	78	56.6	1.29
	B-10-1	R	85	178	CF	0.72	1.00	47	56.6	1.25
	B-11-1	R	85	178	DF	0.91	1.00	97	56.6	1.43
	A-1-1A	R	85	85	DF	0.91	1.00	97	56.6	0.75
	A-1-1B	R	85	115	DF	0.91	1.00	97	56.6	1.13
	A-1-1C	R	85	145	DF	0.91	1.00	97	56.6	1.41
	A-1-1D	R	85	178	DF	0.91	1.00	97	56.6	1.84
	B-1-2A	R	85	85	DF	0.91	2.00	97	56.6	0.83
	B-1-2B	R	85	115	DF	0.91	2.00	97	56.6	1.12
	B-1-2C	R	85	145	DF	0.91	2.00	97	56.6	1.47
	B-1-2D	R	85	178	DF	0.91	2.00	97	56.6	1.86
[79]	B1	R	100	155	HF	0.50	0.75	75.0	20.5	1.10
	C1	R	100	155	HF	0.50	1.25	75.0	21.4	1.15
	D1	R	100	155	HF	0.50	1.75	75.0	21.6	1.24
[71]	F-1	C	D = 250		PIW	0.50	0.50	40.4	20.4	6.75
	F-2	C	D = 250		PIW	0.50	1.00	40.4	21.8	7.73
	F-3	C	D = 250		PIW	0.50	1.50	40.4	21.5	8.00
	F-4	C	D = 250		PIW	0.50	2.00	40.4	21.7	8.61
	F-5	C	D = 250		PIW	0.50	2.50	40.4	20.6	8.74
	F-6	C	D = 250		PIW	0.50	3.00	40.4	22.3	10.18
	F-7	C	D = 250		PIW	0.50	0.50	57.4	21.0	7.36
	F-8	C	D = 250		PIW	0.50	1.00	57.4	20.6	8.10
	F-9	C	D = 250		PIW	0.50	1.50	57.4	20.8	8.95
	F-10	C	D = 250		PIW	0.50	2.00	57.4	21.4	10.27
	F-11	C	D = 250		PIW	0.50	2.50	57.4	22.0	11.35
	F-12	C	D = 250		PIW	0.50	0.50	74.5	20.9	7.67
	F-13	C	D = 250		PIW	0.50	1.00	74.5	20.7	8.84
	F-14	C	D = 250		PIW	0.50	1.50	74.5	20.3	9.99
	F-15	C	D = 250		PIW	0.50	2.00	74.5	21.8	11.25
	F-16	C	D = 250		PIW	0.50	0.50	40.4	21.6	7.30
	F-17	C	D = 250		PIW	0.50	1.00	40.4	22.0	8.40
	F-18	C	D = 250		PIW	0.50	1.50	40.4	20.2	8.06
	F-19	C	D = 250		PIW	0.50	2.00	40.4	20.9	9.23
	F-20	C	D = 250		PIW	0.50	2.50	40.4	21.8	9.72
	F-21	C	D = 250		PIW	0.50	0.50	45.5	24.4	7.77
	F-22	C	D = 250		PIW	0.50	1.00	45.5	26.8	9.44
	F-23	C	D = 250		PIW	0.50	1.50	45.5	25.9	9.37
	F-24	C	D = 250		PIW	0.50	2.00	45.5	26.0	10.74
	F-25	C	D = 250		PIW	0.50	2.50	45.5	25.3	11.06
	F-26	C	D = 250		PIW	0.50	3.00	45.5	25.8	13.06
	F-27	C	D = 250		PIW	0.50	0.50	75.8	26.1	9.83
	F-28	C	D = 250		PIW	0.50	1.00	75.8	27.5	10.78
	F-29	C	D = 250		PIW	0.50	1.50	75.8	26.2	12.26
	F-30	C	D = 250		PIW	0.50	2.00	75.8	26.6	13.49
	F-31	C	D = 250		PIW	0.50	0.50	40.0	14.8	8.08
	F-32	C	D = 250		PIW	0.50	1.00	40.0	25.0	8.91
	F-33	C	D = 250		PIW	0.50	1.50	40.0	24.1	9.26
	F-34	C	D = 250		PIW	0.50	2.00	40.0	23.9	9.72
	F-35	C	D = 250		PIW	0.50	2.50	40.0	24.6	10.47
	F-36	C	D = 250		PIW	0.50	3.00	40.0	24.5	10.70
	F-37	C	D = 250		PIW	0.50	0.50	60.0	24.1	8.39
	F-38	C	D = 250		PIW	0.50	1.00	60.0	24.8	9.78
	F-39	C	D = 250		PIW	0.50	2.00	60.0	24.8	11.41
	F-40	C	D = 250		PIW	0.50	3.00	60.0	23.9	13.58
[80]	RP1	R	100	200	HF	0.50	1.00	37.5	19.8	1.84
	RP3	R	100	200	HF	0.50	3.00	37.5	19.3	1.95
	LsP1	L	100	200	HF	0.50	1.00	37.5	21.0	2.21
	LP1	L	100	200	HF	0.50	1.00	37.5	30.0	2.65
	TsP1	T	100	200	HF	0.50	1.00	37.5	25.0	2.72
	TP1	T	100	200	HF	0.50	1.00	37.5	29.1	2.96
	THsP1	T	100	200	HF	0.50	1.00	37.5	27.3	3.39
	THsP3	T	100	200	HF	0.50	3.00	37.5	33.0	3.69
	THP1	T	100	200	HF	0.50	1.00	37.5	26.0	4.05
	THP3	T	100	200	HF	0.50	3.00	37.5	29.5	4.42
[75]	P20-F1	R	100	200	PIW	0.50	0.30	75.1	22.4	1.88
	P20-F2	R	100	200	PIW	0.50	0.60	75.1	23.3	1.92
	P20-F3	R	100	200	PIW	0.50	0.90	75.1	24.2	2.10
	P20-F4	R	100	200	PIW	0.50	1.20	75.1	25.4	2.27
	P30-F1	R	100	200	PIW	0.50	0.30	75.1	32.4	2.18
	P30-F2	R	100	200	PIW	0.50	0.60	75.1	33.2	2.35
	P30-F3	R	100	200	PIW	0.50	0.90	75.1	34.2	2.52
	P30-F4	R	100	200	PIW	0.50	1.20	75.1	35.0	2.65
	P40-F1	R	100	200	PIW	0.50	0.30	75.1	41.5	2.39
	P40-F2	R	100	200	PIW	0.50	0.60	75.1	42.2	2.52
	P40-F3	R	100	200	PIW	0.50	0.90	75.1	43.0	2.61
	P40-F4	R	100	200	PIW	0.50	1.20	75.1	44.3	2.91
	P50-F1	R	100	200	PIW	0.50	0.30	75.1	52.4	2.31
	P50-F2	R	100	200	PIW	0.50	0.60	75.1	53.9	2.65
	P50-F3	R	100	200	PIW	0.50	0.90	75.1	54.8	2.95
	P50-F4	R	100	200	PIW	0.50	1.20	75.1	55.1	2.99
[46]	1a	R	102	102	PIW	0.50	1.00	26.3	36.3	0.905
	1b	R	102	102	PIW	0.50	1.00	26.3	32.3	0.903
	1c	R	102	102	PIW	0.50	1.00	26.3	36.6	0.893
	2a	R	102	102	PIW	0.50	1.00	38.6	35.0	0.923
	2b	R	102	102	PIW	0.50	1.00	38.6	34.1	0.922
	2c	R	102	102	PIW	0.50	1.00	38.6	35.0	0.915
	3a	R	102	102	PIW	0.50	1.00	52.6	34.1	0.922
	3b	R	102	102	PIW	0.50	1.00	52.6	35.2	0.963
	3c	R	102	102	PIW	0.50	1.00	52.6	33.6	0.903
	4a	R	102	102	PIW	0.50	1.00	77.2	32.3	0.996
	4b	R	102	102	PIW	0.50	1.00	77.2	33.9	0.932
	4c	R	102	102	PIW	0.50	1.00	77.2	32.7	0.959
	5a	R	102	102	PIW	0.50	2.00	52.6	29.7	0.981
	5b	R	102	102	PIW	0.50	2.00	52.6	31.5	0.981
	5c	R	102	102	PIW	0.50	2.00	52.6	31.1	0.986
	6a	R	102	102	PIW	0.50	3.00	52.6	33.7	1.055
	6b	R	102	102	PIW	0.50	3.00	52.6	33.9	1.049
	6c	R	102	102	PIW	0.50	3.00	52.6	35.0	1.050
[50]	RP1	R	100	200	HF	0.50	1.00	37.5	19.8	1.84
	RP3	R	100	200	HF	0.50	3.00	37.5	19.3	1.95
	LsP1	L	100	200	HF	0.50	1.00	37.5	21.0	2.21
	LP1	L	100	200	HF	0.50	1.00	37.5	30.0	2.65
	TsP1	T	100	200	HF	0.50	1.00	37.5	25.0	2.72
	TP1	T	100	200	HF	0.50	1.00	37.5	29.1	2.96
	THsP1	T	100	200	HF	0.50	1.00	37.5	27.3	3.39
	THsP3	T	100	200	HF	0.50	3.00	37.5	33.0	3.69
	THP1	T	100	200	HF	0.50	1.00	37.5	26.0	4.05
	THP3	T	100	200	HF	0.50	3.00	37.5	29.5	4.42
**Minimum value**			**85**	**85**	**-**	**0.5**	**0.30**	**37.5**	**14.8**	**0.75**
**Maximum value**			**250**	**200**	**-**	**1.3**	**6.00**	**156**	**59.0**	**13.58**

^1^ Cross-section shape: R: rectangular; C: circular; L and T: flanged (L- and T-shaped). ^2^ Type of fibers: DF: duoform; RF: round; CF: crimped; HF: hooked; PIW: Plain iron wires.

**Table A2:** Details of the tested SFRC beams of group two (87 specimens).

Reference	Name	Shape ^1^	*x*	*y*	Type of Fibers ^2^	*β*	*ρ_f_*	*l_f_/d_f_*	*f_cu_*	*f_ly_*	*ρ_l_*	*f_ty_*	*ρ_t_*	*T*
			(mm)	(mm)			(%)		(MPa)	(MPa)	(%)	(MPa)	(%)	(kNm)
[79]	B2	R	100	155	HF	0.50	0.75	75.0	20.5	390	0.73	–	–	1.30
	B3	R	100	155	HF	0.50	0.75	75.0	20.5	390	0.73	371	1.14	1.75
	C2-1	R	100	155	HF	0.50	1.25	75.0	21.4	375	0.73	–	–	1.32
	C2-2	R	100	155	HF	0.50	1.25	75.0	21.4	390	0.73	–	–	1.37
	C2-3	R	100	155	HF	0.50	1.25	75.0	21.4	450	1.30	–	–	1.38
	C3-1	R	100	155	HF	0.50	1.25	75.0	21.4	390	0.73	371	0.82	1.91
	C3-2	R	100	155	HF	0.50	1.25	75.0	21.4	390	0.73	371	1.07	2.03
	D2	R	100	155	HF	0.50	1.75	75.0	21.6	390	0.73	–	–	1.43
	D3	R	100	155	HF	0.50	1.75	75.0	21.6	390	0.73	371	1.14	2.27
[81]	T1	R	152	310	HF	1.00	0.50	60.0	40.2	350	1.26	400	1.10	13.95
	T2	R	152	310	HF	1.00	1.00	60.0	40.2	350	1.26	400	1.10	15.67
[82]	T05	R	125	300	HF	1.00	0.50	80.0	24.2	400	1.10	400	2.34	7.50
	T10	R	125	300	HF	1.00	1.00	80.0	26.6	400	1.10	400	2.34	9.00
	T15	R	125	300	HF	1.00	1.50	80.0	25.5	400	1.10	400	2.34	8.50
[44]	LF1	R	85	178	DF	0.50	1.34	97.4	43.1	314	1.35	–	–	2.65
	LF2	R	85	178	DF	0.50	1.91	97.4	42.3	310	0.77	–	–	2.63
	RF1	R	85	178	DF	0.50	0.90	97.4	42.3	314	1.35	368	0.45	2.80
	LF3	R	85	178	DF	0.50	1.34	97.4	41.8	314	1.35	–	–	2.43
	LF4	R	85	178	DF	0.50	1.86	97.4	41.4	368	0.25	–	–	2.31
	RF2	R	85	178	CF	0.50	0.59	100.0	51.3	310	0.77	310	0.77	2.74
	RF3	R	85	178	CF	0.50	0.82	100.0	49.1	310	0.77	310	0.54	2.56
	RF4	R	85	178	CF	0.50	1.09	100.0	46.1	310	0.77	368	0.25	2.60
	RF5	R	85	178	CF	0.50	1.16	100.0	48.6	310	0.77	368	0.18	2.76
	RF6	R	85	178	CF	0.50	0.52	100.0	48.6	368	0.25	310	1.34	2.18
	RF7	R	85	178	CF	0.50	1.11	100.0	46.1	368	0.25	310	0.77	2.18
	RF8	R	85	178	CF	0.50	1.42	100.0	44.9	368	0.25	368	0.45	2.67
	RF9	R	85	178	CF	0.50	1.61	100.0	47.5	368	0.25	368	0.25	2.63
	RF10	R	85	178	CF	0.50	0.84	100.0	49.1	339	0.48	310	1.34	2.74
	LF6	R	85	178	CF	0.50	1.59	100.0	48.4	339	0.48	–	–	2.46
	LF7	R	85	178	CF	0.50	0.95	100.0	48.2	310	1.15	–	–	2.76
	LF8	R	85	85	CF	0.50	1.06	100.0	48.2	310	1.61	–	–	1.02
	LF9	R	85	145	CF	0.50	1.42	100.0	44.9	310	0.94	–	–	1.83
[57]	A-0.5	R	300	300	PIW	0.50	0.50	37.6	25.8	380	0.70	380	0.79	27.34
	A-1.0	R	300	300	PIW	0.50	1.00	37.6	21.4	380	0.70	380	0.79	29.01
	A-1.5	R	300	300	PIW	0.50	1.50	37.6	28.0	380	0.70	380	0.79	34.67
	B-1.0	R	300	300	PIW	0.50	1.00	37.6	21.4	380	1.05	380	1.18	36.46
	C-1.0	R	300	300	PIW	0.50	1.00	37.6	21.4	380	1.40	380	1.57	40.86
[74]	B2	R	100	200	HF	0.50	0.60	75.2	31.2	250	1.57	250	0.00	1.41
	B3	R	100	200	HF	0.50	1.20	75.2	40.1	250	1.57	250	0.00	1.74
	B5	R	100	200	HF	0.50	0.60	75.2	38.9	250	1.57	250	0.35	2.29
	B6	R	100	200	HF	0.50	1.20	75.2	35.6	250	1.57	250	0.35	2.84
[58]	R40C-F1	R	100	200	PIW	0.50	0.30	75.1	40.1	500	1.01	500	1.68	5.56
	R40C-F2	R	100	200	PIW	0.50	0.60	75.1	41.1	500	1.01	500	1.68	5.69
	R40C-F3	R	100	200	PIW	0.50	0.90	75.1	42.0	500	1.01	500	1.68	5.73
	R40C-F4	R	100	200	PIW	0.50	1.20	75.1	43.3	500	1.01	500	1.68	5.82
	R40L-F1	R	100	200	PIW	0.50	0.30	75.1	41.3	500	1.57	500	0.85	4.11
	R40L-F2	R	100	200	PIW	0.50	0.60	75.1	42.2	500	1.57	500	0.85	4.19
	R40L-F3	R	100	200	PIW	0.50	0.90	75.1	43.4	500	1.57	500	0.85	4.23
	R40L-F4	R	100	200	PIW	0.50	1.20	75.1	44.1	500	1.57	500	0.85	4.23
	R40T-F1	R	100	200	PIW	0.50	0.30	75.1	41.5	500	0.57	500	1.51	3.85
	R40T-F2	R	100	200	PIW	0.50	0.60	75.1	42.8	500	0.57	500	1.51	3.93
	R40T-F3	R	100	200	PIW	0.50	0.90	75.1	43.1	500	0.57	500	1.51	3.98
	R40T-F4	R	100	200	PIW	0.50	1.20	75.1	43.9	500	0.57	500	1.51	4.02
[83]	RL-F1	R	100	200	PIW	0.50	0.30	75.1	35.2	500	1.57	500	0.02	2.01
	RL-F2	R	100	200	PIW	0.50	0.60	75.1	37.0	500	1.57	500	0.02	2.27
	RL-F3	R	100	200	PIW	0.50	0.90	75.1	37.7	500	1.57	500	0.02	2.61
	RL-F4	R	100	200	PIW	0.50	1.20	75.1	38.4	500	1.57	500	0.02	2.82
	RT-F1	R	100	200	PIW	0.50	0.30	75.1	33.9	500	0.14	500	2.51	1.75
	RT-F2	R	100	200	PIW	0.50	0.60	75.1	34.4	500	0.14	500	2.51	2.31
	RT-F3	R	100	200	PIW	0.50	0.90	75.1	35.0	500	0.14	500	2.51	2.57
	RT-F4	R	100	200	PIW	0.50	1.20	75.1	35.3	500	0.14	500	2.51	2.69
[50]	RL1	R	100	200	HF	0.50	1.00	37.5	17.0	415	1.57	–	–	2.41
	RL3	R	100	200	HF	0.50	3.00	37.5	16.4	415	1.57	–	–	2.73
	RR1	R	100	200	HF	0.50	1.00	37.5	19.0	415	1.57	344	0.75	2.73
	RR3	R	100	200	HF	0.50	3.00	37.5	16.9	415	1.57	344	0.75	3.15
[84]	R50C-FI	R	100	200	PIW	0.50	0.30	75.1	51.0	432	1.57	432	1.51	6.67
	R50C-F2	R	100	200	PIW	0.50	0.60	75.1	51.8	432	1.57	432	1.51	6.76
	R50C-F3	R	100	200	PIW	0.50	0.90	75.1	52.5	432	1.57	432	1.51	6.84
	R50C-F4	R	100	200	PIW	0.50	1.20	75.1	53.9	432	1.57	432	1.51	6.93
	R50L-F1	R	100	200	PIW	0.50	0.30	75.1	51.1	432	1.57	432	0.80	5.22
	R50L-F2	R	100	200	PIW	0.50	0.60	75.1	52.1	432	1.57	432	0.80	5.30
	R50L-F3	R	100	200	PIW	0.50	0.90	75.1	53.4	432	1.57	432	0.80	5.39
	R50L-F4	R	100	200	PIW	0.50	1.20	75.1	54.1	432	1.57	432	0.80	5.47
	R50T-FI	R	100	200	PIW	0.50	0.30	75.1	52.6	432	0.57	432	1.51	5.77
	R50T-F2	R	100	200	PIW	0.50	0.60	75.1	53.2	432	0.57	432	1.51	5.82
	R50T-F3	R	100	200	PIW	0.50	0.90	75.1	54.1	432	0.57	432	1.51	5.90
	R50T-F4	R	100	200	PIW	0.50	1.20	75.1	55.5	432	0.57	432	1.51	5.99
[76]	L08F40V3	R	150	200	HF	0.50	0.30	40.0	33.4	460	0.67	460	0.55	4.58
	L08F40V6	R	150	200	HF	0.50	0.60	40.0	31.3	460	0.67	460	0.55	5.68
	L08F55V3	R	150	200	HF	0.50	0.30	54.5	31.0	460	0.67	460	0.55	4.94
	L08F55V6	R	150	200	HF	0.50	0.60	54.5	30.9	460	0.67	460	0.55	5.87
	L08F67V3	R	150	200	HF	0.50	0.30	66.7	32.7	460	0.67	460	0.55	4.92
	L08F67V6	R	150	200	HF	0.50	0.60	66.7	29.5	460	0.67	460	0.55	5.88
	L08F80V3	R	150	200	HF	0.50	0.30	80.0	31.9	460	0.67	460	0.55	4.85
	L08F80V6	R	150	200	HF	0.50	0.60	80.0	30.0	460	0.67	460	0.55	5.49
	L12F40V3	R	150	200	HF	0.50	0.30	40.0	31.7	460	1.51	460	0.55	6.01
	L12F80V3	R	150	200	HF	0.50	0.30	80.0	31.6	460	1.51	460	0.55	6.25
**Minimum value**			**85**	**85**	**-**	**0.50**	**0.30**	**37.5**	**16.4**	**250**	**0.14**	**250**	**0.00**	**1.02**
**Maximum value**			**300**	**310**	**-**	**1.00**	**3.00**	**100**	**55.5**	**500**	**1.61**	**500**	**2.51**	**40.86**

^1^ Cross-section shape: R: rectangular; C: circular; L and T: flanged (L- and T-shaped). ^2^ Type of fibers: DF: duoform; RF: round; CF: crimped; HF: hooked; PIW: Plain iron wires.
